# Transcriptional regulation of Ligase IV by an intronic regulatory element directs thymocyte development

**DOI:** 10.1038/s41435-025-00353-3

**Published:** 2025-09-05

**Authors:** Matthew D. Estrada, Christopher J. Gebhardt, Mariam A. Salem, Christina N. Rau, Kruthika Sharma, Rebecca A. Glynn, Craig H. Bassing, Eugene M. Oltz, Patrick L. Collins

**Affiliations:** 1 Department of Microbial Infection and Immunity, The Ohio State University, Columbus, OH USA; 2 Molecular, Cellular and Developmental Biology Graduate Program, The Ohio State University, Columbus, OH USA; 3 Department of Pathology and Laboratory Medicine, Children’s Hospital of Philadelphia, Perelman School of Medicine, University of Pennsylvania, Philadelphia, PA USA

**Keywords:** Immunogenetics, Gene regulation, Gene regulation in immune cells, Lymphocytes

## Abstract

Double-strand breaks represent the most dangerous form of DNA damage, and in resting cells, these breaks are sealed via the non-homologous end joining (NHEJ) factor Ligase IV (LIG4). Excessive NHEJ may be genotoxic, necessitating multiple mechanisms to control NHEJ activity. However, a clear mechanism of transcriptional control for them has not yet been identified. Here, we examine mechanisms governing *Lig4* transcription in mammals, finding that most tissues maintain very low levels of LIG4 production. Select tissues upregulate LIG4, employing different strategies for genomic regulation. In developing lymphocytes, the *Lig4* locus is devoid of long-range chromatin contacts; instead, its expression and role in immune development depend upon a promoter-proximal intronic regulatory element. Deletion of the Lig4 intronic regulatory element results in thymocyte-specific loss of Lig4 upregulation, defects in lymphocyte development, and altered antigen receptor rearrangement. Our findings show the NHEJ gene, *Lig4*, is transcriptionally controlled to support stage-specific function concurrent with programmed DSBs. Moreover, we provide an example of how DNA cis-regulatory elements very close to a promoter can have substantial transcriptional effects.

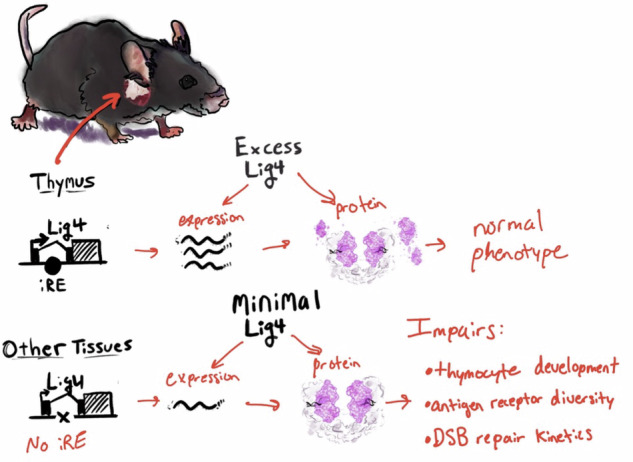

## Introduction

The most dangerous form of DNA damage is thought to be double-strand breaks (DSBs), which may stem from endogenous sources, such as programmed DNA breaks during lymphocyte development or replication-induced breaks, as well as from exogenous sources like ionizing radiation. How well a specific cell type responds to these DSBs varies. For example, developing lymphocytes in the thymus must efficiently perform non-homologous end joining (NHEJ) to repair their developmentally programmed DSBs, but they are also highly vulnerable to DSBs caused by ionizing radiation [1]. Other examples of cell types with high levels of endogenous DSBs include bone marrow stem cells [2], intestinal crypts [3], and cortical neurons [4]. Consequently, individuals with deficiencies in the NHEJ repair gene *Ligase IV* (*Lig4*) exhibit severe neuronal, digestive, growth, hematopoietic, and immunological defects [2, 5–7]. Understanding the DNA damage sensitivity of these organs is crucial in medical treatments such as radiation and chemotherapy for cancer, as well as for developing targeted gene therapies for patients with NHEJ deficiencies.

LIG4 is a core component of the NHEJ process that involves (i) identifying DNA ends via the Ku70/80 complex, which recruits the DNA-PKcs complex, (ii) forming a long-range DNA-PKcs-LIG4-XRCC4-XLF synaptic complex, (iii) processing DNA ends as needed, (iv) evicting DNA-PKcs to form a short-range LIG4-XRCC4-XLF synaptic complex, and finally (v) ligating DNA ends via LIG4 activity [8]. Thus, LIG4 is strictly necessary for canonical NHEJ because of both non-catalytic activity that stabilizes DSBs and allows alternative repair pathways [9], and direct catalytic activity responsible for NHEJ.

LIG4-dependent mutagenesis can lead to errors such as deletions, insertions, and structural variation, and thus, regulation is critical for preventing genomic damage. One way to avoid excessive or inappropriate DNA repair involves controlling LIG4’s nuclear import and stability through binding with X-ray repair cross-complementing 4 (XRCC4) [10, 11]. Moreover, current models based on fibroblast siRNA knockdown experiments suggest that even undetectably low levels of *Lig4* expression are sufficient for repair [12]. As will be discussed below, most tissues maintain a very low level of Lig4 expression, and it remains unclear whether there is any selective in vivo pressure to maintain these baseline LIG4 expression levels.

Adaptive lymphocytes and neuronal development have an essential need for LIG4-mediated repair [13]. In neurons, DSBs are thought to arise as a natural byproduct of their high metabolic activity and transcription, as well as resolution of topoisomerase lesions [14]. In contrast, in developing lymphocytes, DSBs are programmed through the process known as variable-diversity-joining rearrangement (V(D)J) recombination. During V(D)J recombination, the recombination-activating gene (RAG) 1/2 enzymes induce DSBs at antigen receptor (AgR) gene segments. LIG4 seals these breaks, creating functional B cell receptor (BCR) or T cell receptor (TCR) genes [15]. Since the recombination process occurs on the chromosomal DNA, all daughter clones from a newly developed lymphocyte inherit the newly created TCRs or BCR genes, allowing adaptive immunity.

Given that LIG4 can be mutagenic when overactive but is required in all tissues, we investigated the cell types expressing high and low levels of LIG4. In this study, we observed tissue-specific upregulation of LIG4 in thymocytes, consistent with its role in V(D)J recombination. Unlike many gene loci, the *Lig4* region is remarkably simple, containing a single intronic regulatory element (iRE) that correlates with gene expression levels in lymphocytes. We show that the deletion of this iRE results in measurable differences in tissue-specific expression, development of immune cells, and radiosensitivity.

## Materials/subjects and methods

### Online data mining

All online data mining was current as of August 2024. Bulk RNA-seq was downloaded from the Immunological Genome Project (ImmGen) [16] and visualized using their RNA-seq skyline (https://rstats.immgen.org/Skyline/skyline.html) and iPhone application web portals (https://www.immgen.org/ImmGenApps.html). Immgen ATAC-seq [17] data were processed and visualized using the UCSC genome browser (https://genome.ucsc.edu/) [18]. Whole-body mouse single-cell RNA-seq data were acquired from the Tabula Muris project [19] and visualized using the associated CellXGene interface (https://tabula-muris.ds.czbiohub.org/). De-identified pediatric cancer data were acquired from the St. Jude cloud [20] (https://www.stjude.cloud/), filtered for newly diagnosed hematological cases, and then plotted using GraphPad Prism. Paired acute lymphoblastic leukemia (ALL) cell line RNA-seq transcriptome and total proteome data originated from Leo et al. [21], and were plotted via the FORALL [22] website (https://proteomics.se/forall/).

### Western blots

Cells were resuspended in lysis buffer (50 mM Tris Base pH 8, 150 mM NaCl, 1% v/v Triton-X) with protease inhibitor (Cell Signaling: 5871S) for 30 min on ice. After centrifugation at 16,000 × *g* for 20 min at 4 °C, the supernatant was collected. Laemmli buffer was added, and the mixture was boiled for 5 min. Proteins were then separated in 4–20% SDS-PAGE (Bio-Rad) and transferred onto an Immun-Blot PVDF membrane (Bio-Rad). Blocking of the membrane was achieved by soaking it for 1 h in Tris-Buffered Saline Tween (TBST) (20 mM Tris pH 7.5, 150 mM NaCl, 0.1% Tween-20) containing 5% non-fat milk. Primary antibodies were added for overnight incubation (Ligase IV, Cell Signaling:14649 and GAPDH, Santa Cruz: sc25778). The next day, after washing three times with 0.1% TBST, secondary antibodies were added for a 1-h incubation (Anti-Rabbit HRP, Cell Signaling: 7074). Following three washes with 0.1% TBST, blots were scanned with an X-ray machine and visualized on film.

### Knock out mouse creation

Mice were created at the Children’s Hospital of Philadelphia (CHoP) Research Institute Transgenic Core using Cas9 RNP nucleofection. RNP oligos were designed using CCTOP [23] to target the Lig4-iRE. These oligos, along with crRNA and Cas9, were purchased from IDT. RNP complexes were formed ex vivo and then electroporated into 0.5-day gestation zygotes using a Gene Pulser Electroporator and 1 mm cuvettes. The resulting mice were screened for the Lig4-iRE deletion by PCR, and two lines with independent mutations were chosen. The mouse lines were backcrossed to C57BL/6 for three generations in a specific pathogen-free facility before creating homozygotic lines. For all experiments involving animals, researchers were blind to genotype until after data acquisition and analysis. All animal studies were reviewed and approved by The Ohio State University Animal Care and Use Program.

### RNA extraction from tissues and qRT-PCR

For expression analysis, 8-week-old mice were euthanized according to institutional guidelines. The spleen, pancreas, heart, thymus, liver, brain, eye, and colon tissues were placed in separate Precellys homogenizer tubes (Bertin) containing 1 mL of TRIzol (ThermoFisher, 15596026). The precise location of each tissue and method of extraction were done per Johnson et al. [24]. Samples were homogenized using a Precellys Evolution Touch Homogenizer set to 5500 RPM (spleen, pancreas, heart, thymus, liver, brain, colon) or 6500 RPM (eye) for two 20-s cycles. The soluble fraction was then pipetted into correspondingly labeled RNase-free tubes and stored at −80 °C. For bone marrow, cells were isolated by centrifugation [25], filtered, and depleted of red blood cells using a brief ACK lysis before storage at −80 °C. RNA was extracted from TRIzol aliquots using Monarch Total RNA Miniprep Kit (NEB, T2010) with an on-column DNase step. 1 μg of RNA was either reserved as a no reverse transcription (RT) control or processed into cDNA using M-MuLV Reverse Transcriptase (NEB, M0253S) with a poly A primer (IDT). Relative abundances of Beta-2 microglobulin (B2M) and *Lig4* transcripts were determined by qPCR using a SYBR green 2× master mix and analyzed using the 2^(-ΔΔCT) method [26]. Statistical analyses were performed using unpaired Student’s *t*-tests.

### Cell culture

Splenocytes were grown in RPMI media supplemented by the manufacturer with 4.5 g/L D-Glucose, L-glutamine, and sodium pyruvate (Gibco, 11995065). Media was completed with 10% heat-inactivated fetal bovine serum (FBS, Sigma F4135), 100 U/mL penicillin/streptomycin (Gibco, 15140122), and 55 μM β-Mercaptoethanol (Gibco, 21985023). B cells were isolated by magnetic depletion, stained with Cell Trace Violet, and resuspended at 1 × 10^6^/mL in fresh RPMI supplemented with either diluted LPS (Sigma) or LPS and IL-4 (Sigma). Cells were cultured for 72 h before analyzing.

### Mixed bone marrow chimeras and flow cytometry

CD45.1 JAXBoy mice were purchased age- and sex-matched from Jackson Laboratory and allowed to acclimate to the colony for two weeks before use. Following acclimation, CD45.1 and CD45.2 Lig4-iRE knockout animals were euthanized according to institutional protocols, bone marrow was isolated, and the cells were mixed at a 50:50 ratio. For recipients, C57BL/6 mice were lethally irradiated (10 Gy) with a Radsource 2000 X-ray device and retro-orbitally injected with 5 × 10^6^ mixed donor cells. Correct mixing was verified by flow cytometry. After six to nine weeks, animals were euthanized, and cells were stained with CD19-APC-H7 (BD: 560143), B220-APC (Invitrogen: 17-0452-82), CD43-FITC (Biolegend: 121206), CD24-PE (Biolegend: 138504), c-Kit-BUV395 (BD: 584001), CD11b-BV650 (Biolegend: 101259), CD3-Biotin (Biolegend: 100244), Ly6C-Biotin (Biolegend: 108404), Ter119-Biotin (Biolegend: 116204), StrepA-V450 (BD: 560797), Ghost-viability-V510 (Cell Signaling: 59863S), IgM-PE-Cy7 (Invitrogen: 25-5790-82), and IgD-PerCP5.5 (Biolegend: 405710) for the bone marrow panel. For analyzing thymocytes, cells were stained with CD4-APC (Invitrogen: 17-0042-81), CD8-FITC (BD: 553031), CD44-PE (Invitrogen: 12-0441-81), CD25-PECy5.5 (Invitrogen: 35-0251-80), TCRγ-BV421 (Biolegend: 118120), Ghost-viability-V510 (Cell Signaling: 59863S), Ly6C-Biotin (Biolegend: 108404), CD11b-BV650 (Biolegend: 101259), CD19-Biotin (Biolegend: 115504), CD11c-Biotin (Biolegend: 117304), NK1.1-Biotin (Biolegend: 108704), and StrepA-AF700 (Invitrogen: S21383) and analyzed using a Cytek Aurora flow cytometer. For class switching experiments, flow cytometry was done with IgG1-APC (eBioscience: 17-4714-81).

### T cell receptor beta repertoire analysis

DN thymocytes from WT and Lig4-iRE^−/−^ mice 6–8 weeks of age were purified by negative lineage selection using Biotin MACs magnetic columns (Miltenyi Biotec: 130-090-858). Antibodies are as follows: Ter119-Biotin (Biolegend: 116204), NK1.1-Biotin (Biolegend: 108704), CD11c-Biotin (Biolegend: 117304), CD11b-Biotin (Biolegend: 101203), CD19-Biotin (Biolegend: 115504), followed by labeling with Anti-Biotin MicroBeads. RNA was extracted from the purified cells and made into libraries using a commercial kit (NEBNext Immune Sequencing Kit), according to the manufacturer’s instructions. Sequenced libraries were aligned using MixCR (4.7.0) with the settings neb-mouse-rna-xcr-umi-nebnext. Further analysis was performed using the R package immunarch (1.0.0).

### Image stream analysis of DNA damage foci

Lig4-iRE and WT mice were irradiated (2 or 6 Gy) with a Radsource 2000 X-ray device. Thymocytes were isolated and stained with a fixable viability dye (Ghost V560) followed by fixation and permeabilization with the Foxp3/Transcription Factor Staining Buffer Set (eBioscience) according to the supplier’s manual. H2Ax and phopsho-H2Ax were stained overnight (anti-H2Ax-AF594, anti-phospho-H2Ax-APC both Biolegend). Cells were analyzed on an ImageStream®X Mk II Imaging Flow Cytometer (Cytek) in EDF mode at 60× magnification. Analysis of phospho-H2Ax damage foci was performed using the IDEAS® software (version 6.2, Amnis).

## Results

### LIG4 is transcriptionally upregulated in progenitor and precursor lymphocytes

**Fig. 1:**
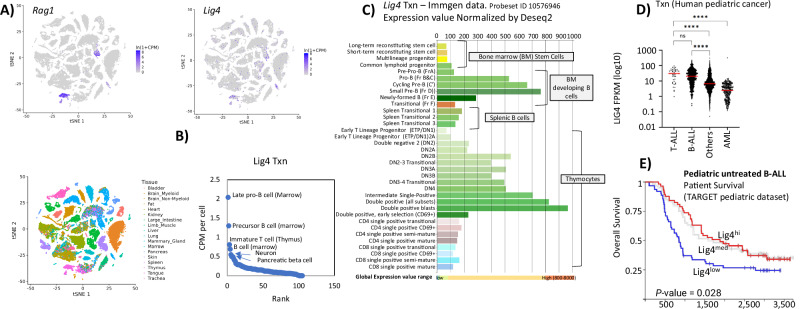
Expression of LIG4. **A** tSNE representation of mouse single-cell RNA-seq, taken from a dataset that processed eighteen different organs (https://tabula-muris.ds.czbiohub.org/). The blue pseudocolor plots show normalized counts for *Rag1* and *Lig4* transcripts. On the bottom, the cells are colored by originating organ. **B** Each cell type was extracted from data in (**A**), and then plotted for Lig4 CPM expression and gene rank. The top six *Lig4*-expression ranked cell clusters are labeled. **C** Expression of *Lig4*, taken as a snapshot from the Immgen microarray data browser (https://www.immgen.org/). Cell type is listed to the left, expression values are shown on top and bottom. **D** Pediatric cancer expression analysis from the St. Jude’s web portal (https://pecan.stjude.cloud/). ****p < 0.0001, Student’s *T*-test. **E** Pediatric B acute lymphoblastic leukemia (B ALL) survival data from the Xena web browser (https://xenabrowser.net/) TARGET PAN-CAN dataset. *P*-values show *χ*^2^ statistics.

LIG4 is not only required for lymphocyte and neuronal development but is also needed for cells responding to unprogrammed DNA DSBs. To evaluate *Lig4* expression across cell types, we analyzed whole-mouse single-cell RNA-seq data from the Chan-Zuckerberg CellxGene project [27]. Sporadic low-level *Lig4* transcription was observed in most tissues, with the highest transcription in *Rag1*^*+*^ clusters of developing lymphocytes from the Thymus and Bone Marrow (Fig. 1A). Specifically, progenitor B (pro-B) cells (mean CPM per cell 2.04), precursor-B (pre-B) cells (mean CPM per cell 1.3), and immature thymocytes (mean CPM per cell 0.81) have the highest *Lig4* expression. The next highest levels were in neurons and pancreatic beta cells, which had four-fold lower *Lig4* expression compared to pro-B cells (mean CPM 0.49 and 0.40, respectively) (Fig. 1B). Thymus-specific single-cell RNA-seq (Fig. S1A) and spatial RNA-seq (Fig. S1B) data showed Lig4 expression was highest in CD4+ CD8+ thymocytes and the thymic cortex.

Given the elevated levels of *Lig4* transcripts in *Rag1*^+^ immune progenitor clusters, we examined expression across lymphocytes using data from the Immunological Genomes (ImmGen) project consortium [28]. Bulk RNA-seq showed that *Lig4* was highly expressed in Hardy fraction D [29] bone marrow, which are precursor (pre)-B cells that express a pre-BCR, and developing thymocytes (Fig. 1C). Additionally, elevated expression levels were observed in other cell stages that require V(D)J recombination for antigen receptor joining like pro-B cells but returned to homeostatic levels in splenic B cells and single positive T cells (Fig. 1C). We conclude that *Lig4* transcription is upregulated during programmed V(D)J recombination and rapidly silenced after repair.

Acute lymphoblastic leukemia (ALL) most closely resembles precursor and progenitor B- and T-lymphocytes [30], and the malignancies’ evolution depends upon NHEJ [31]. Based on this similarity, we examined whether Lig4 is upregulated in cancers that phenotypically resemble these cell types. We analyzed pediatric RNA-seq datasets from St. Jude’s online web portal [20]. Expression of *Lig4* is especially high in B- and T-ALL when compared to other childhood cancers, whereas acute myeloid leukemia (AML) samples expressed the lowest levels of *Lig4* (Fig. 1D). More differentiated ALL types that may phenotypically resemble B cell precursors, such as the BCR-ABL and RUNX1 subtypes, expressed the highest levels of *Lig4* expression (Fig. S1C) [32]. However, *Lig4* transcription was also highly predictive of overall survival in ALL patients (Fig. 1E), but the result could not be separated from the effects of the underlying subtype (Fig. S1C). In contrast, *Lig4* expression was low in all types of AML, with the poorly differentiated RUNX1/RUNX1T1 fusion subtypes [33] expressing modest levels of *Lig4*. Thus, *Lig4* expression tracks with progenitor and precursor lymphocyte differentiation and is mirrored in cancerous equivalents that resemble pre- and pro-T and B cells.

### Identification of a putative LIG4 intronic regulatory element (iRE)

**Fig. 2:**
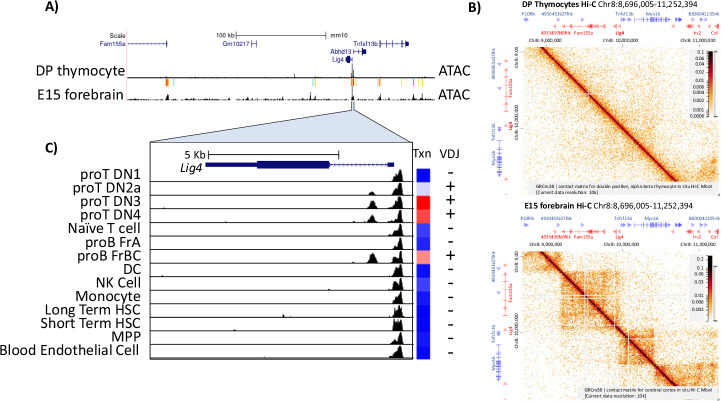
Chromatin landscape of the *Lig4* locus. **A** UCSC genome browser snapshot of the LIG4 locus and surrounding genes. The Top track shows relative genomic scale and gene location. Genes are represented as thick dark blue lines for coding exons, and thin lines for introns. Bottom tracks represent ATAC-seq data from double-positive thymocytes (immgen) and E15 forebrain (ENCODE), with MACs2 peak calling above the tracks. **B** HiGlass snapshots of Hi-C data from the 4D nucleosome project (https://4dnucleome.org/), which shows data derived from double-positive thymocytes (top) and E15 forebrain (bottom). Genes are separated by strand, with forward genes shown in blue and reverse genes in red. Cross-tissue ATAC-seq analysis, derived from the ImmGen consortium, showing the LIG4 region highlighted in (**C**). The heatmap to the right shows *Lig4* expression, with red being the highest and blue being the lowest. On top, thin bars are untranslated regions, thick bars are translated exons, and narrow lines are introns.

We have shown that *Lig4* transcription levels are developmentally controlled, which for many genes is dependent upon cis-enhancers and related regulatory elements (REs). Thus, we evaluated ATAC-seq data from the Immgen [28] and ENCODE consortia [34]. As shown in Fig. 2A, the *Lig4* regulatory landscape is remarkably simple in double-positive (DP) thymocytes, in which this gene is expressed at high levels. There are two accessible gene promoters (*Lig4*, and neighboring *Abhd13*) surrounded by a megabase (Mb) region with no other major thymocyte, or B cell (data not shown) accessible sites (Fig. 2A). For comparison, we examined ENCODE-derived data for embryonic day 15 (E15) forebrain, where *Lig4* supports proper neuronal development [13]. This analysis revealed distal accessible peaks that were absent in DP thymocytes (Fig. 2A). Thus, one possibility is that developing neurons, but not lymphocytes, utilize distal accessibility for *Lig4* control, as is seen with most cis-regulatory elements [35]. Consistent with that speculation, analysis of public Hi-C chromatin contact data from the 4D consortium [36] showed no distal contacts above background levels in DP thymocytes. In contrast, the E15 forebrain, a stage when LIG4 is needed for development, exhibited topologically associated domain (TAD) structure and loop calls. These observations suggest that distal regulatory interactions play no significant role in Lig4 upregulation in developing thymocytes, contrasting with neuronal control in the forebrain.

We next investigated the *Lig4* promoter region for genomic features linked to tissue-specific expression. The mouse *Lig4* gene has a single, short, non-coding exon, a single intron, and a single coding exon. Immgen ATAC data revealed an accessible element within the *Lig4* intron that correlates with cell-type-selective expression. Immune cell types with high *Lig4* transcription, like pro-B cells and double-negative (DN) thymocytes, have intronic accessibility. In contrast, immune cell types with reduced *Lig4* expression showed accessibility restricted to the transcription start site (Fig. 2C). Thus, we hypothesized that elevated *Lig4* expression within developing lymphocytes is controlled by an intronic regulatory element (iRE).

### Discovery of an early lymphocyte-specific regulatory element devoted to Lig4 regulation

**Fig. 3:**
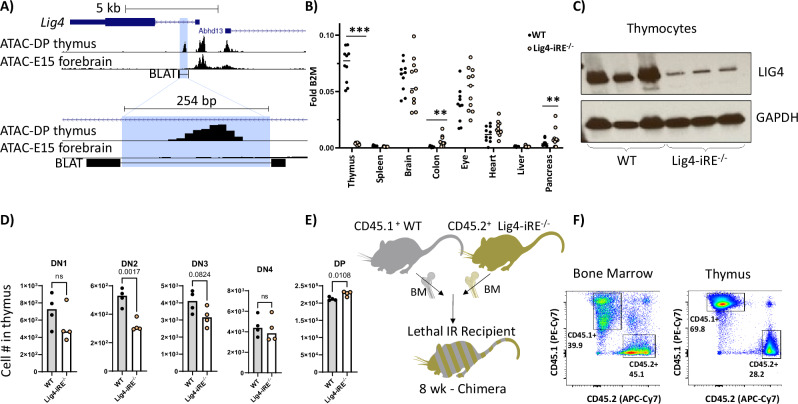
Creation and initial phenotyping of Lig4-iRE-knockout mice. **A** UCSC genome browser snapshots, as in Fig. 2A. The blue highlight shows the Lig4-iRE deletion region. Bottom BLAT track shows sequenced region (thick bars). **B** Whole thymus, spleen, brain, colon, eye, heart, liver, and pancreas were analyzed for *Lig4* RNA versus B2M control RNA. Dots are biological replicates, and statistics are a *T*-test. **p < 0.01, ***p < 0.0001. **C** Whole thymic extracts were analyzed for Ligase IV protein from WT mice and mice with an intronic deletion. Each lane is a different mouse. **D** Absolute numbers of indicated thymocytes in 9-week males. Student’s *T*-test *P*-values are indicated above bars. Representative flow plots are shown in (**B**). **E** Diagram showing a competitive bone marrow chimera experiment. **F** Flow cytometry of mixed bone marrow chimera experiments showing percentage of CD45.1 WT and CD45.2 Lig4-iRE knockout thymocytes or bone marrow cells. Data are quantified in (**C**).

To test the function of the Lig4 intronic regulatory element, we deleted the precursor and progenitor lymphocyte-specific 254 bp accessible region, termed the Lig4-iRE (Fig. 3A). We verified Lig4-iRE deletion by sequencing two independent knockout lines with distinct deletional scars (Fig. S3A). Lig4-iRE knockout mice were viable, exhibited normal birth frequencies, and showed no overt motor defects, strongly indicating NHEJ is intact in the developing nervous system. We then examined *Lig4* expression in a set of tissues that express a range of Lig4 transcript levels (Fig. 3B). In wild type (WT) animals, thymus had the highest *Lig4* expression of all the tissues, which was strictly dependent on the Lig4-iRE (Fig. 3B). Notably, a slight but statistically significant increase in *Lig4* transcription was observed in the colon and pancreas of Lig4-iRE knockout mice (Fig. 3B). Western blot analysis of thymus extracts confirmed a notable reduction in protein levels in Lig4-iRE knockout animals compared to WT animals (Fig. 3C). Collectively, these data identify a regulatory element that drives high levels of LIG4 in the thymus but not within other tissues, consistent with the distinct regulatory mechanisms observed in E15 forebrain.

We next tested whether elevated Lig4 expression, which was dependent on the iRE, was necessary for normal thymocyte development. When comparing nine-week-old mice under homeostatic conditions, we observed significantly reduced numbers of DN2 cells from Lig4-iRE knockout thymuses, relative to WT (Fig. 3D & representative flow plots in Fig. S3B), and increased numbers of DP cells. To test for an intrinsic role of the Lig4-iRE in lymphoid development, we created bone marrow chimeras by transplanting a 50:50 ratio of CD45.1^+^ WT bone marrow cells to CD45.2^+^ Lig4-iRE knockout bone marrow cells into 10 Gy lethally irradiated CD45.2^+^ recipient mice (Diagram on Fig. 3E). We found that nine weeks after transplantation, relative to bone marrow, there were significantly less CD45.2+ Lig4-iRE knockout cells in the thymus (Fig. 3F). Relative to the parent CD45 gate, there were significantly increased proportions of Lig4-iRE knockout CD45.2^+^ DN2 cells and decreased proportions of Lig4-iRE knockout CD45.2^+^ DN4 cells (Figs. 3F & S3C). Together, these findings demonstrate that *Lig4* is over-expressed in thymocytes dependent upon an iRE and that deletion of this regulatory element significantly disrupts thymocyte development, specifically by decreasing overall DN2 cell numbers in the thymus and skewing the progression to DN4 cells.

### Lig4 upregulation in thymocytes is needed for normal V(D)J recombination

Programmed DNA breaks in precursor and progenitor lymphocytes stem from V(D)J recombination and RAG activity, which is repaired by LIG4-dependent NHEJ. We hypothesized that downregulation of Lig4 due to iRE deletion could disrupt the pre-selection T cell receptor beta (TCRβ) repertoire, potentially affecting lymphocyte development. To evaluate this, we analyzed RNA sequences to examine TCRβ recombination events specifically in a pre-selection repertoire. Pre-selection repertoires allow direct observation of the diversity generated during V(D)J recombination without the confounding effects of thymic selection for functional receptors. Moreover, analyzing RNA identifies which V(D)J rearrangements are actively transcribed in cells.

**Fig. 4:**
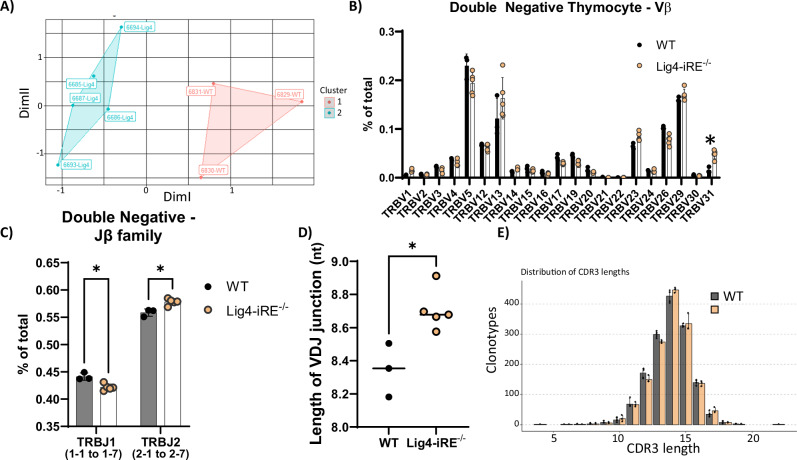
T cell receptor beta recombination in DN cells. **A** DN thymocytes were processed for TCRβ repertoire and plotted via multi-dimensional scaling (MDS) analysis with K-means clustering. Each point represents a biological replicate, and relative genotypes of Lig4-iRE knockout or WT cells are labeled next to the data points. **B** Analysis of Vβ use from DN cells. Dots are biological replicates, and statistics are a *T*-test. *p < 0.01. **C** Jβ use summarized by all J1 segments vs all J2 segments. **D** Average length of nucleotides added to the TCRβ V(D)J junctions in biologically independent replicates. Statistics are a *T*-test. *p < 0.01 **E** Distribution of CDR3 lengths in WT versus Lig4-iRE knockout DN cells.

To isolate these TCRβ sequences, we magnetically sorted DN thymocytes (CD4^-^CD8^-^CD11b^-^CD19^-^CD11c^-^NK1.1^-^GR1^-^) and used a 5′CAGE approach to prepare the libraries. Using k-means clustering based on antibody receptor sequences, we identified distinct clustering between WT and Lig4-iRE knockout DN thymocytes (Fig. 4A), suggesting differences in recombination outcomes. We next evaluated V and J segment usage. Notably, Lig4-iRE knockout DN cells had significantly greater usage of TRBV31 (Fig. 4B) and TRBJ2 family (TRBJ2-1 through TRBJ2-7) segments (Fig. 4C) compared to WT cells, indicating altered segment selection or junctional processing. Notably, TRBV31 is unique among TRBV segments, as it is located 5′ of the DJ segments, is the closest in genomic distance, and undergoes recombination by inversion.

We hypothesized that reduced *Lig4* expression might result in a prolonged timeframe for the enzyme terminal deoxynucleotidyl transferase (TdT) to add more nucleotides to DSB ends during V(D)J recombination [37]. Consistent with this, we observed a 0.2-nucleotide increase in the average length of V(D)J junctions in Lig4-iRE knockout CDR3 regions compared to WT DN controls (Fig. 4D, E). Thus, deletion of the Lig4-iRE resulted in thymus-specific downregulation of *Lig4*, longer CDR3 junction lengths, and changes in thymus cellularity.

### Lig4 regulation in thymocytes is associated with radiosensitivity

**Fig. 5:**
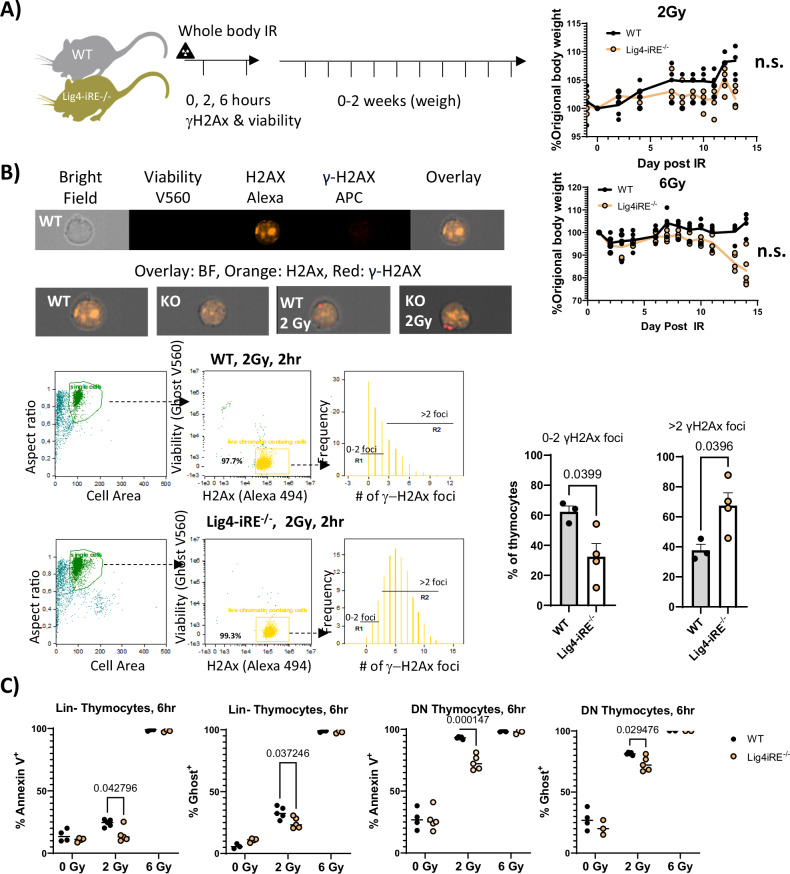
Response to exogenous radiation in Lig4-iRE-knockout mice. **A** Schematic illustration of the whole-body irradiation experimental setup. Animals were given 0, 2, or 6 Gy doses of whole-body X-ray irradiation, and analyzed at 2 and 6 h by flow cytometry, or up to two weeks by weighing. **B** Image stream flow cytometry showing H2A.x, γ-H2A.x, and viability in thymocytes after 0 or 2 Gy of whole-body X-ray irradiation. Histograms show foci count for γ-H2A.x in Ghost^-^ H2A.x^+^ irradiated cells, and quantification across replicates is shown on the right. **C** Lineage negative (Lin-) and double-negative (Lin-CD4-CD8-) thymocytes were stained with Annexin V and Ghost Dye. *P*-values are Student’s *T*-test, and significance values are shown on top.

We next investigated how WT and Lig4-iRE knockout mice respond to exogenous DNA damage caused by radiation. We first randomized sex and weight-matched WT and Lig4-iRE knockout mice between cages, and then irradiated them with 0, 2, or 6 Gy of X-ray irradiation (Fig. 5A diagram). Two hours post-irradiation, thymocytes were processed for imaging flow cytometry using Ghost, total H2A.x, and γH2A.x stains (Fig. 5B). We observed bright γH2A.x foci in the irradiated cells, but few to no foci in the live WT and Lig4-iRE knockout thymocytes at homeostasis (Fig. 5B, Supplementary Fig. 5A, B). At 2 h post-irradiation, the majority of WT thymocytes had 0–2 γH2A.x foci, whereas Lig4-iRE knockout thymocytes predominantly had more than 2 γH2A.x foci, suggesting slower repair.

Five to six hours post-irradiation, apoptosis was measured using Ghost and Annexin V stains, separately evaluating DN (CD4^-^CD8^-^Lineage^-^) and Lineage^-^ (Lin^-^) thymocytes due to intrinsically high rates of apoptosis in the former. At baseline, there was no significant change in Ghost^+^ or Annexin^+^ staining in Lig4-iRE knockout Lin^-^ cells compared to WT Lin^-^ cells (Fig. 5C). 6 Gy of irradiation was sufficient to kill almost all thymocytes, regardless of genotype. However, after 2 Gy irradiation, we observed a significant protective effect from the Lig4-iRE deletion in both DN and Lin^-^ thymocytes (Fig. 5C & Supplementary Fig. 5C). In a separate experiment, animals were irradiated and monitored for weight recovery over two weeks. No significant difference in weight recovery was observed between WT and Lig4-iRE knockout mice (Fig. 5A). Collectively, these results suggest that Lig4-iRE knockout thymocytes have slower DSB repair, as evidenced by increased γH2A.x foci at 2 h after 2 Gy irradiation, along with slightly slower kinetics of cellular apoptosis relative to WT thymocytes.

### Phenotype of Lig4-iRE-deficient B cells

**Fig. 6:**
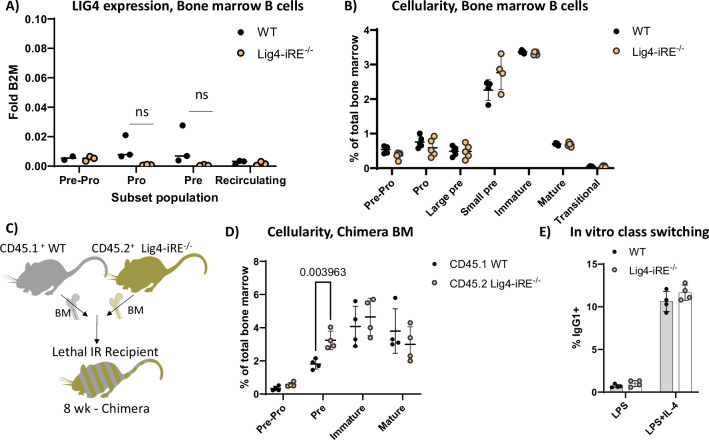
B cell phenotype of Lig4-iRE-knockout mice. **A** Sorted bone marrow populations were analyzed for *Lig4* RNA versus B2M control RNA. Dots are biological replicates. **B** Absolute percentages of indicated Hardy fractions in 9-week males. Each dot is an individual replicate, and all differences are non-significant by a student’s *T*-test. **C** Diagram showing a competitive bone marrow chimera experiment. **D** Flow cytometry of mixed bone marrow chimera experiments. Dots are biological replicates, and statistics are a *T*-test. Representative flow cytometry dot plots are shown in Fig. S6A. **E** In vitro class-switch assay. Isolated B cells were cultured for four days with LPS ± IL-4, and class switching to IgG1 was determined by flow cytometry.

Since *Lig4* is highly expressed in pro- and pre-B cells during their development in the bone marrow, we analyzed the effect of the Lig4-iRE on B cell development. Bone marrow B cells (defined as live, B220^+^Lineage^-^) were sorted into pro-B (B220^mid^CD43^+^), pre-B (B220^mid^CD43^-^), and recirculating B (B220^high^) subsets for Lig4 transcription analysis via qRT-PCR (Fig. 6A). WT pro- and pre-B cells exhibited a non-significant trend toward higher *Lig4* transcript levels compared to Lig4-iRE knockout cells. Notably, average *Lig4* expression in WT bone marrow B cell subsets was lower than total thymic levels (compare Fig. 6A to Fig. 3B). When using flow cytometry to compare WT and Lig4-iRE, there were no significant differences in cellularity (Fig. 6B). However, when we examined a competitive case using mixed bone marrow chimeras at 9 weeks post-reconstitution, we found an accumulation in the percentage of CD45.2^+^B220^+^CD43^+^ (Lig4-iRE) progenitor bone marrow B cells, compared to CD45.1+ (WT) controls (Fig. 6C, D).

We did not observe *Lig4* upregulation in the spleen when compared to other tissues (see Fig. 3B). However, it remained possible that there could be a class switching defect in Lig4-iRE knockout mice, since NHEJ plays a role in class-switch recombination [38]. To evaluate, we first cultured splenic B cells with LPS (proliferation control) or LPS + IL-4 (to induce class switching). After four days, CD138^-^ proliferating cells (cell trace violet^dim^) were analyzed for IgG production. We did not observe any significant difference between WT and knockout conditions (Fig. 6E). Together, *Lig4* upregulation occurs in bone marrow progenitor B cells, but overall B cell expression differences and phenotypic impacts on cellularity are less obvious when compared to thymocytes.

### Transcriptional regulation of LIG4 depends upon E2A and HEB

**Fig. 7:**
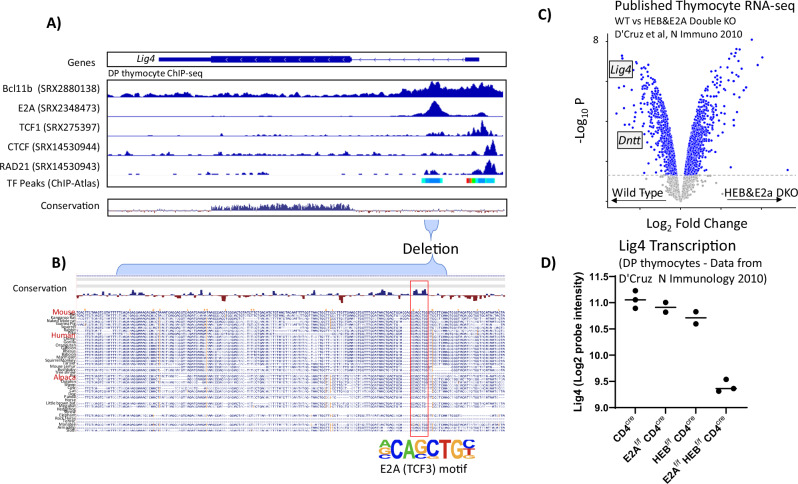
The transcription factors HEB&E2A govern *Lig4* transcription. **A** Integrated genome viewer (IGV) snapshot that shows published thymocyte ChIP-seq data, which was processed via the ChIP-Atlas web portal (SRX numbers are unique identifiers for data origination and publication). On the top is genomic localization and gene position, with thin bars being untranslated regions, and thick bars representing the translated exon. The middle histograms show published ChIP-seq data, with each histogram scaling from minimum to maximum. A combined peak track showing all ChIP-seq follows. The bottom track shows vertebrate conservation, which was derived from the UCSC genome browser conservation data, and the above blue represents conserved sequences. **B** Zoom-in of the deleted region. Multi-species conservation is indicated with select species that are used to produce antibodies listed in red. **C** Data shows gene expression taken from [49], which evaluated sorted double-positive thymocyte gene expression in CD4^cre^ animals (controls), or also with E2A ^flox/flox^ and HEB^Flox/flox^ **D** Lig4 probe expression in all genotypes from D’Cruz et al.

Lastly, we sought to understand the molecular mechanisms endowing the Lig4-iRE with its cell-type-specific enhancer activity. We first analyzed public thymocyte ChIP-seq data to determine which transcription factors bind to the *Lig4* promoter and intronic region (Fig. 7A). Since expression and cellularity phenotypes were more pronounced in thymocytes, we analyzed thymic peaks from the ChIP-Atlas database [39], which showed the binding of thymocyte lineage commitment transcription factors Bcl11b and E2A to the Lig4-iRE. We also observed that TCF1, CTCF, and RAD21 bound to the promoter region, but not significantly at the intronic regulatory element. Closer analysis of transcription factor binding motifs in the deleted intronic region revealed a conserved motif for E2A DNA sequences (Fig. 7B). In the thymus, it is thought that E2A and related HEB have redundant functions in regulating lymphocyte development and differentiation [40–42]. To discern if both factors are needed, we took advantage of microarray expression data derived from E2A and HEB double knockouts, versus each knockout alone [43]. The E2A-HEB double knockout datasets demonstrated a loss of *Lig4* expression relative to WT DP thymocytes, whereas knockout of just E2A or just HEB had no effect (Fig. 7C). Together, we conclude that the stage-specific transcription factors E2A and HEB directly bind the Lig4-iRE and are responsible for subsequent transcriptional upregulation.

## Discussion

Here, we demonstrate that *Lig4* is transcriptionally low in most tissues, except in progenitor and precursor lymphocytes. During thymocyte development, the transcription factors E2A and HEB bind to a *Lig4* intronic regulatory element, driving an increase in expression. However, this high level of expression is rapidly attenuated following differentiation. While all cells require some LIG4 production for efficient NHEJ, most maintain minimal *Lig4* transcription during homeostasis. In developing thymocytes, the inability to upregulate LIG4, via deletion of the *Lig4* intronic regulatory element, results in thymic cellularity defects, including a competitive disadvantage and altered antigen receptor composition. After exposure to 2 Gy of irradiation, Lig4-iRE knockout thymocytes had delayed kinetics of DSB repair and slower apoptosis. One potential reason for the reduced apoptosis observed in Lig4-iRE knockout thymocytes post-challenge is that unrepaired DSBs may activate cell cycle halt and/or senescence before apoptosis by replication, immune activity, and/or oxidative stress, which awaits further testing. Together, these results show that *Lig4* transcriptional regulation contributes to the antigen receptor repertoire diversity during development and DSB repair.

Deletion of the Lig4-iRE had a modest in vivo phenotype, including altered thymic cellularity, increased CDR3 junction lengths, and a shift in B cell development that was observable after a competitive bone marrow transfer. Given the redundancy of DNA repair pathways, it is possible that other factors, such as those involved in alternative end-joining pathways or homologous recombination, for example, may partially compensate for the absence of LIG4 upregulation during lymphopoiesis. Transcriptional regulation may be the only mechanism by which LIG4 activity is controlled. For example, unlike other NHEJ repair factors, LIG4 is incapable of nuclear translocation without XRCC4, which regulates its nuclear localization, abundance, and phase condensate formation [10, 11]. Having multiple mechanisms of control is essential to restrain the genotoxic effect of LIG4. Future research should investigate additional mechanisms controlling LIG4 activity and their therapeutic potential in diseases where LIG4 is dysregulated, such as ALL. Indeed, the expression of NHEJ proteins has largely been assumed to be constitutive, and exploiting the underlying pathways is an unexplored avenue for treating leukemia.

Additionally, excessive LIG4 activity may interfere with replication processes. Supporting this idea, during cell cycling, replication forks may collapse when encountering lesions such as TOP1-protein DNA complexes or trapped PARP1 [44]. In such instances, single-end DSBs are generated, which must be repaired through ATM-activated homologous recombination rather than potentially toxic NHEJ [35, 45]. Hence, ATM-deficient cells are susceptible to TOP1 inhibitors like camptothecin and topotecan, but not Atm^-/-^Lig4^-/-^ cells or Atm^-/-^Lig4-catalytic domain mutants [35]. These findings reinforce the idea that multiple layers of LIG4 regulation, including transcriptional control, nuclear shuttling by XRCC4, and involvement of ATM, are necessary to protect the genome from toxic NHEJ activity.

We also found elevated *Lig4* expression in acute lymphoblastic leukemias and corresponding cell lines, which has implications for interpreting DNA damage research conducted in these systems. For example, prior research compared LIG4 hypomorphs made in a human pre-B ALL cell line termed NALM6 [46], and their translocation and mutational potential following designer nuclease damage. These studies, and subsequent reviews [47], concluded that LIG4-dependent NHEJ causes translocations in humans, while alternative-NHEJ causes translocations in mouse germinal center B cells [38, 48]. Our results suggest a new possibility, that extreme LIG4 levels in B ALL cell lines like NALM6 influence the response to nuclease damage. Targeting the Lig4-iRE or modulating its activity might serve as a novel therapeutic approach for ALL and other malignancies where NHEJ is dysregulated. By integrating chromatin and transcriptional data, this study offers new insights into the molecular and transcriptional programs governing DSB responsiveness in both healthy and transformed cells.

Together, our findings reveal that Lig4 transcription in lymphocytes is tightly controlled by a proximal intronic regulatory element, enabling stage-specific upregulation during antigen receptor assembly. This regulation, mediated by E2A and HEB, is important for maintaining thymocyte cellularity and shaping antigen receptor diversity. Additionally, the study highlights the significant impact of localized cis-regulatory elements on tissue-specific gene expression, with implications for both normal development and leukemia pathogenesis.

## Supplementary information

Supplementary Figure Legends

Supplementary Figures

## Data Availability

The datasets generated and/or analyzed during the current study are available in the GEO repository and available from the corresponding author on reasonable request.
