# Supplementary material for: Transcriptional regulation of Ligase IV by an intronic regulatory element directs thymocyte development
Source: Genes Immun. 2025 Sep 5;26(5):509–18. doi: 10.1038/s41435-025-00353-3 (PMC12527937; doi:10.1038/s41435-025-00353-3)
Supplement: Supplementary file 1 — Supplementary Figure Legends [file 41435_2025_353_MOESM1_ESM.docx]

# Supplemental Figure Legends

## Figure S1. Expression of LIG4 across AML and ALL subtypes

A. tSNE representation of mouse thymus single cell RNA-seq CellXGene data (https://tabula-muris.ds.czbiohub.org/). The blue pseudocolor plots show normalized counts for *Cd8a, CD4,* and *Lig4* transcripts. B. Visum spatial RNA-seq of mouse thymus from the CellXGene project. Spot expression pseudocolor is consistent with panel A. C. Snapshot of data from the St. Jude’s web portal (<https://pecan.stjude.cloud/>), as in figure 1D. Data are separated into box and whisker plots for different ALL and acute myeloid leukemia (AML) subtypes.

## Figure S3. Related to Figure 3.

**A.** DNA sequence for two independent Lig4-iRE knockout lines. Blue represents cloned sequence (top). On bottom is the cloned sequence, and in black is deleted region. **B.** Thymocytes were subjected to surface staining and flow cytometry for the indicated genotypes. Replicates were quantified in figure 3D. **C.** Mixed bone marrow chimera showing percentage of CD45.1 WT and CD45.2 Lig4-iRE knockout thymocytes or bone marrow cells. Dots are biological replicates and statistics are a T test, * p < 0.05. Representative flow plots are shown in Figure 3F. **D.** Mixed bone marrow chimera showing thymocytes as a percent of the parent CD45 gate. Dots are biological replicates and statistics are a T test, * p < 0.05. Representative flow cytometry dot plots are shown in figure S3E. **E.** Flow Cytometry of thymocyte cell surface staining of mixed bone marrow chimera experiments.

## Figure 5S. Related to Figure 5.

**A.** Image stream flow cytometry showing H2A.x, γ-H2A.x, and viability in thymocytes after 0, or 2 Gy of whole-body X-ray irradiation. Cropped data were used for panel B in figure 5. **B.** Histograms show foci count for γ-H2A.x in Ghost^-^ H2A.x^+^ cells in non-irradiated thymocytes, with descriptive statistics below graphs. Representative examples from the R3 gate are shown on right. **C.** The Lineage negative (Lin-) and double negative (Lin-CD4-CD8-) thymocytes were stained with Annexin V and a Ghost Dye and measured by flow cytometry. Data are quantified in Figure 5C.

## Figure S6. Representative flow cytometry plot, related to Figure 6.

Mixed bone marrow chimera animals were subjected to surface staining and flow cytometry for the indicated genotypes. Replicates were quantified in figure 6D.
