# Supplementary material for: Transcriptional regulation of Ligase IV by an intronic regulatory element directs thymocyte development
Source: Genes Immun. 2025 Sep 5;26(5):509–18. doi: 10.1038/s41435-025-00353-3 (PMC12527937; doi:10.1038/s41435-025-00353-3)
Supplement: Supplementary file 2 — Supplementary Figures [file 41435_2025_353_MOESM2_ESM.pdf]

Figure S1

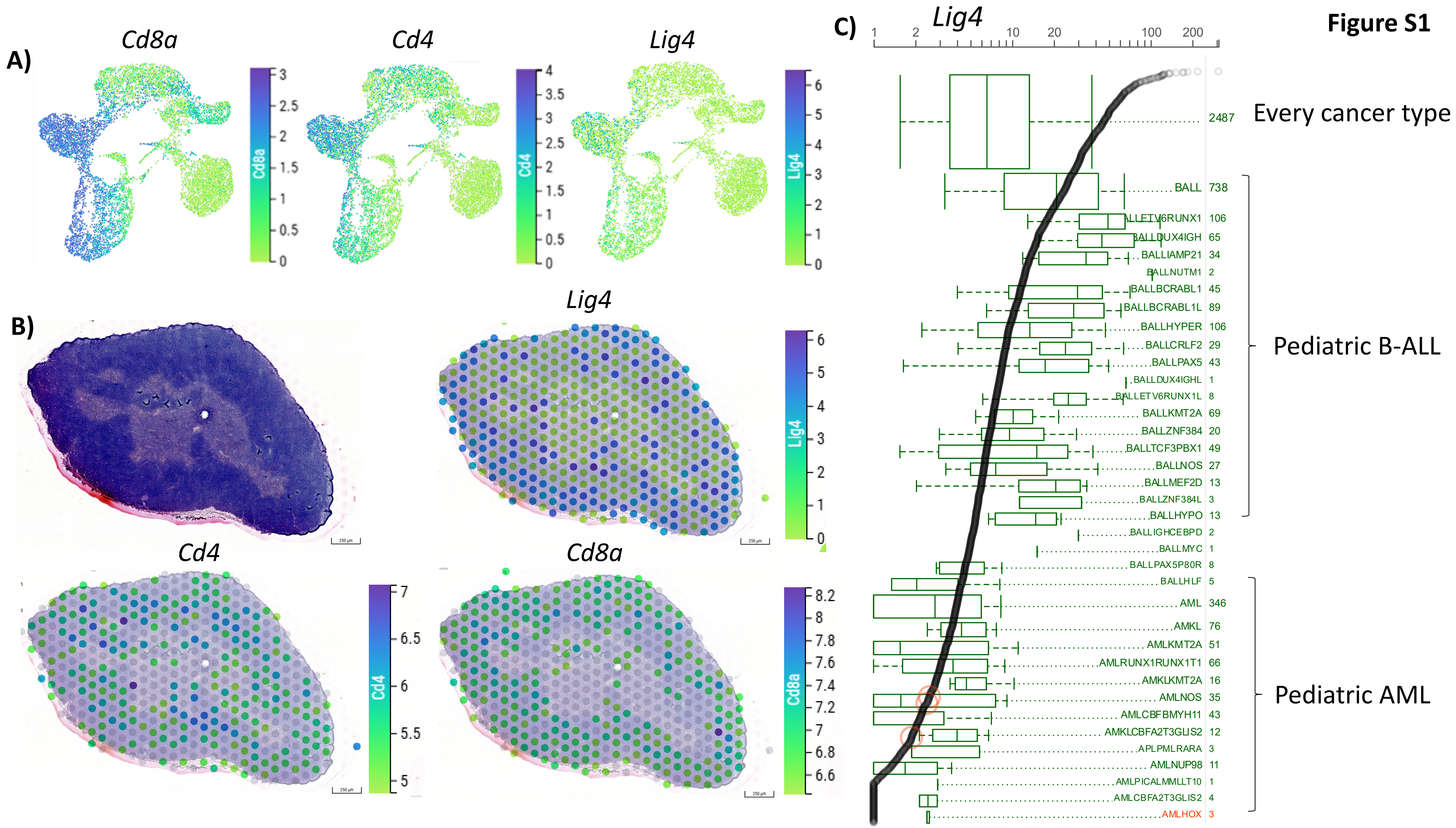
A)
Cd8a
Cd4
Lig4
B)
Lig4
Cd4
Cd8a
C)
Lig4
1 2 10 20 100 200
Every cancer type 2487
BALL 738
BALLETV6RUNX1 106
BALLDUX4IGH 65
BALLIAMP21 34
BALLNUTM1 2
BALLBCRABL1 45
BALLBCRABL1L 89
BALLHYPER 106
BALLCRLF2 29
BALLPAX5 43
BALLDUX4IGHL 1
BALLETV6RUNX1L 8
BALLKMT2A 69
BALLZNF384 20
BALLTCF3PBX1 49
BALLNOS 27
BALLMEF2D 13
BALLZNF384L 3
BALLHYPO 13
BALLIGHCEBPD 2
BALLMYC 1
BALLPAX5P80R 8
BALLHLF 5
Pediatric B-ALL
AML 346
AMKL 76
AMLKMT2A 51
AMLRUNX1RUNX1T1 66
AMKLKMT2A 16
AMLNOS 35
AMLCBFBMYH11 43
AMKLCBFA2T3GLIS2 12
APLPMLRARA 3
AMLNUP98 11
AMLPICALMMLLT10 1
AMLCBFA2T3GLIS2 4
AMLHOX 3
Pediatric AML

Figure S3

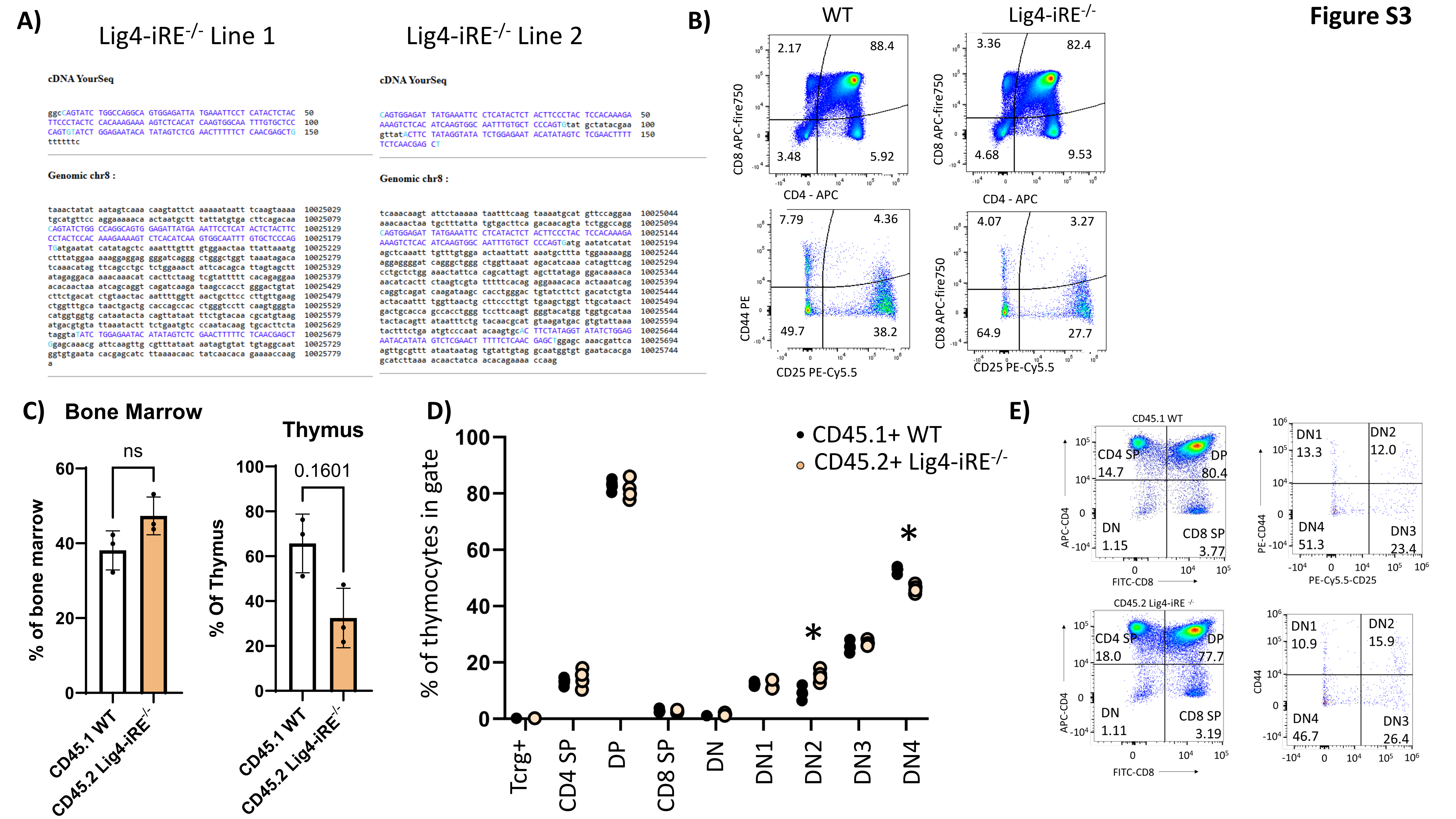
A)
Lig4-iRE-/- Line 1
cDNA YourSeq
ggcCAGTATC TGGCCAGGCA GTGGAGATTA TGAAATTCCT CATACTCTAC 50
TTCCCTACTC CACAAAGAAA AGTCTCACAT CAAGTGGCAA TTTGTGCTCC 100
CAGTGTATCT GGAGAATACA TATAGTCTCG AACTTTTTCT CAACGAGCTG 150
ttttttc
Genomic chr8 :
taaactatat aatagtcaaa caagtattct aaaaataatt tcaagtaaaa 10025029
tgcatgttcc aggaaaaaca actaatgctt tattatgtga cttcagacaa 10025079
CAGTATCTGG CCAGGCAGTG GAGATTATGA AATTCCTCAT ACTCTACTTC 10025129
CCTACTCCAC AAAGAAAAGT CTCACATCAA GTGGCAATTT GTGCTCCCAG 10025179
TGatgaatat catatagctc aaatttgttt gtggaactaa ttattaaatg 10025229
ctttatggaa aaaggaggag gggatcaggg ctgggctggt taaatagaca 10025279
tcaaacatag ttcagcctgc tctggaaact attcacagca ttagtagctt 10025329
atagaggaca aaacaaacat cacttctaag tcgtattttt cacagaggaa 10025379
acacaactaa atcagcaggt cagatcaaga taagccacct gggactgtat 10025429
cttctgacat ctgtaactac aattttggtt aactgcttcc cttgttgaag 10025479
ctggtttgca taactgactg caccagccac ctgggtcctt caagtgggta 10025529
catggtggtg cataatacta cagttataat ttctgtacaa cgcatgtaag 10025579
atgacgtgta ttaaatactt tctgaatgtc ccaatacaag tgcacttcta 10025629
taggtaTATC TGGAGAATAC ATATAGTCTC GAACTTTTTC TCAACGAGCT 10025679
Ggagcaaacg attcaagttg cgtttataat aatagtgtat tgtaggcaat 10025729
ggtgtgaata cacgagcatc ttaaaacaac tatcaacaca gaaaaccaag 10025779
a
Lig4-iRE-/- Line 2
cDNA YourSeq
CAGTGGAGAT TATGAAATTC CTCATACTCT ACTTCCCTAC TCCACAAAGA 50
AAAGTCTCAC ATCAAGTGGC AATTTGTGCT CCCAGTGtat gctatacgaa 100
gttatACTTC TATAGGTATA TCTGGAGAAT ACATATAGTC TCGAACTTTT 150
TCTCAACGAG CT
Genomic chr8 :
tcaaacaagt attctaaaaa taatttcaag taaaatgcat gttccaggaa 10025044
aaacaactaa tgctttatta tgtgacttca gacaacagta tctggccagg 10025094
CAGTGGAGAT TATGAAATTC CTCATACTCT ACTTCCCTAC TCCACAAAGA 10025144
AAAGTCTCAC ATCAAGTGGC AATTTGTGCT CCCAGTGatg aatatcatat 10025194
agctcaaatt tgtttgtgga actaattatt aaatgcttta tggaaaaagg 10025244
aggaggggat cagggctggg ctggttaaat agacatcaaa catagttcag 10025294
cctgctctgg aaactattca cagcattagt agcttataga ggacaaaaca 10025344
aacatcactt ctaagtcgta tttttcacag aggaaacaca actaaatcag 10025394
caggtcagat caagataagc cacctgggac tgtatcttct gacatctgta 10025444
actacaattt tggttaactg cttcccttgt tgaagctggt ttgcataact 10025494
gactgcacca gccacctggg tccttcaagt gggtacatgg tggtgcataa 10025544
tactacagtt ataatttctg tacaacgcat gtaagatgac gtgtattaaa 10025594
tactttctga atgtcccaat acaagtgcAC TTCTATAGGT ATATCTGGAG 10025644
AATACATATA GTCTCGAACT TTTTCTCAAC GAGCTggagc aaacgattca 10025694
agttgcgttt ataataatag tgtattgtag gcaatggtgt gaatacacga 10025744
gcatcttaaa acaactatca acacagaaaa ccaag
B)
WT
Lig4-iRE-/-
CD8 APC-fire750
CD4 - APC
2.17
88.4
3.48
5.92
3.36
82.4
4.68
9.53
CD44 PE
CD25 PE-Cy5.5
7.79
4.36
49.7
38.2
4.07
3.27
64.9
27.7
C)
Bone Marrow
ns
% of bone marrow
CD45.1 WT
CD45.2 Lig4-iRE-/-
Thymus
0.1601
% Of Thymus
D)
CD45.1+ WT
CD45.2+ Lig4-iRE-/-
% of thymocytes in gate
Tcrg+
CD4 SP
DP
CD8 SP
DN
DN1
DN2
DN3
DN4
E)
CD45.1 WT
CD4 SP
14.7
DP
80.4
DN
1.15
CD8 SP
3.77
APC-CD4
FITC-CD8
DN1
13.3
DN2
12.0
DN4
51.3
DN3
23.4
PE-CD44
PE-Cy5.5-CD25
CD45.2 Lig4-iRE -/-
CD4 SP
18.0
DP
77.7
DN
1.11
CD8 SP
3.19
DN1
10.9
DN2
15.9
DN4
46.7
DN3
26.4
CD44

Figure S5

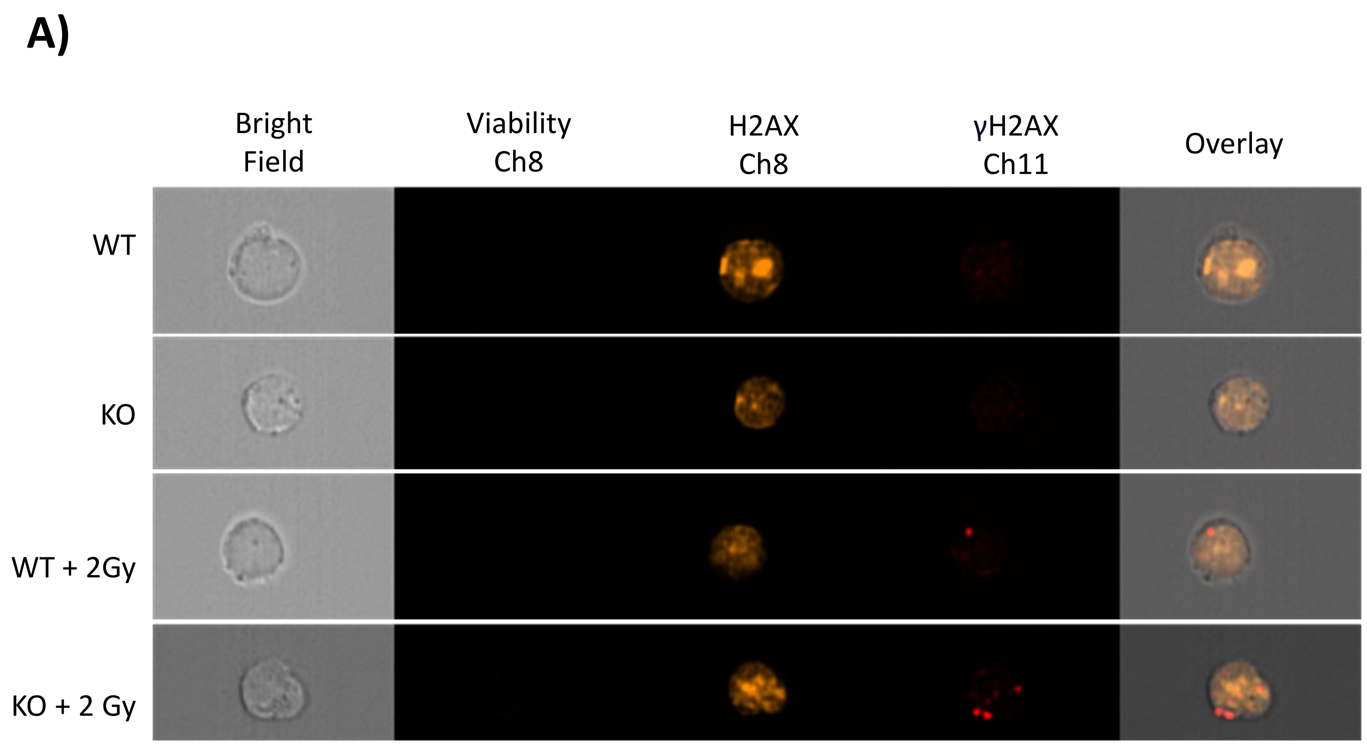

A)
Bright Field
Viability Ch8
H2AX Ch8
γH2AX Ch11
Overlay
WT
KO
WT + 2Gy
KO + 2 Gy

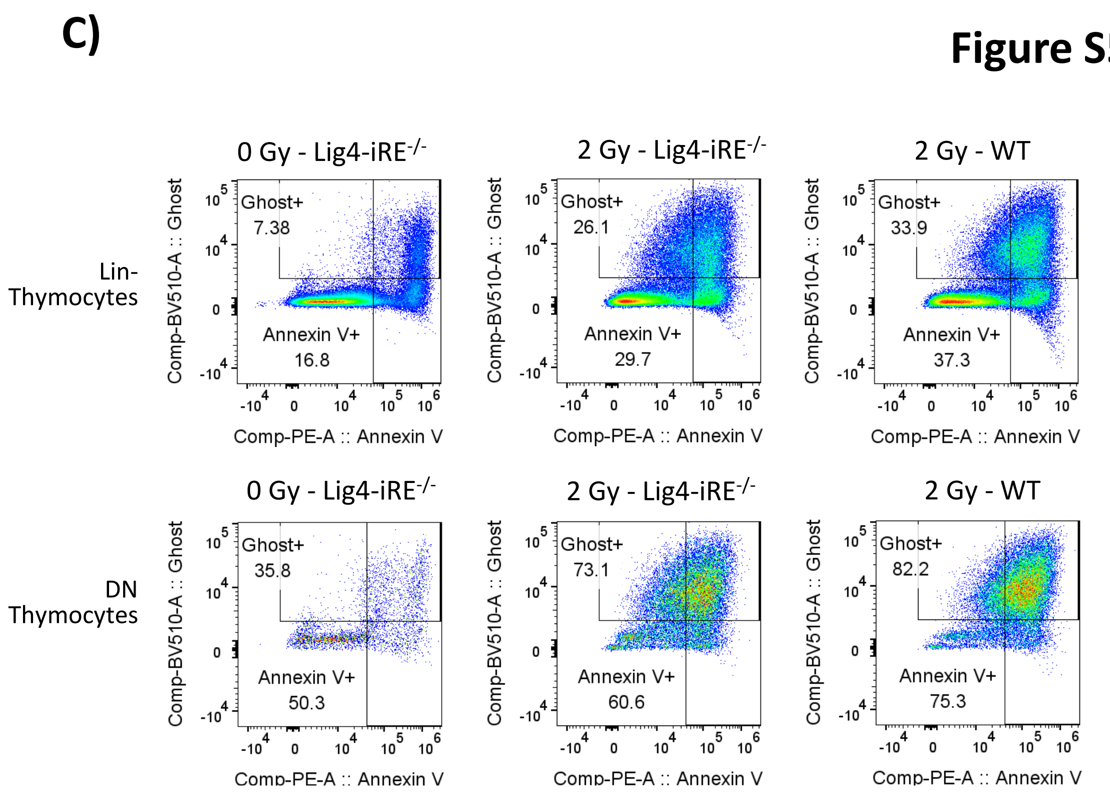

C)
Lin- Thymocytes
DN Thymocytes
0 Gy - Lig4-iRE-/-
2 Gy - Lig4-iRE-/-
2 Gy - WT
Ghost+
7.38
26.1
33.9
35.8
73.1
82.2
Annexin V+
16.8
29.7
37.3
50.3
60.6
75.3
Comp-BV510-A :: Ghost
Comp-PE-A :: Annexin V

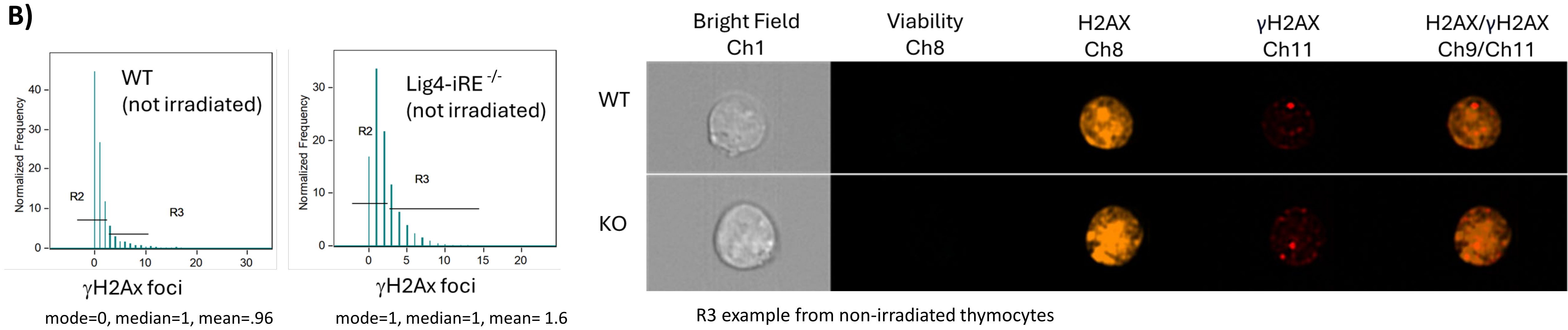

B)
WT (not irradiated)
Lig4-iRE -/- (not irradiated)
Normalized Frequency
R2
R3
γH2Ax foci
mode=0, median=1, mean=.96
mode=1, median=1, mean= 1.6
Bright Field Ch1
Viability Ch8
H2AX Ch8
γH2AX Ch11
H2AX/γH2AX Ch9/Ch11
WT
KO
R3 example from non-irradiated thymocytes

Figure S6

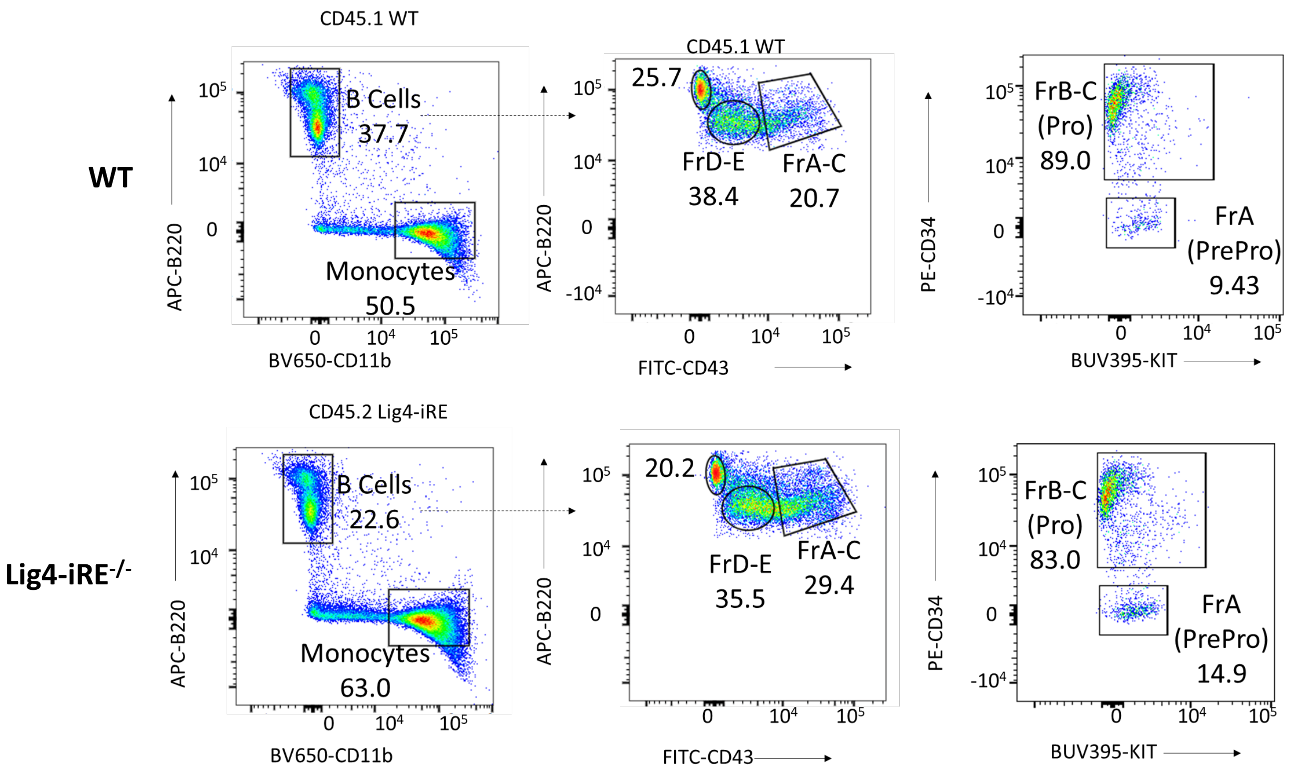

CD45.1 WT
WT
B Cells
37.7
Monocytes
50.5
APC-B220
BV650-CD11b
CD45.1 WT
25.7
FrD-E
38.4
FrA-C
20.7
APC-B220
FITC-CD43
FrB-C
(Pro)
89.0
FrA
(PrePro)
9.43
PE-CD34
BUV395-KIT
CD45.2 Lig4-iRE
Lig4-iRE-/-
B Cells
22.6
Monocytes
63.0
APC-B220
BV650-CD11b
20.2
FrD-E
35.5
FrA-C
29.4
APC-B220
FITC-CD43
FrB-C
(Pro)
83.0
FrA
(PrePro)
14.9
PE-CD34
BUV395-KIT
